# Diet as a system: an observational study investigating a multi-choice system of moderately restricted low-protein diets

**DOI:** 10.1186/s12882-016-0413-5

**Published:** 2016-12-07

**Authors:** Giorgina Barbara Piccoli, Marta Nazha, Irene Capizzi, Federica Neve Vigotti, Stefania Scognamiglio, Valentina Consiglio, Elena Mongilardi, Marilisa Bilocati, Paolo Avagnina, Elisabetta Versino

**Affiliations:** 1 Department of Clinical and Biological Sciences, University of Torino, Torino, Italy; 2 Nephrologie, Centre Hospitalier Le Mans, 72000 Le Mans, France; 3 SS Nephrology, Department of Clinical and Biological Sciences, ASOU San Luigi, University of Torino, Torino, Italy; 4 SCDU Urology, Department of Oncology, ASOU San Luigi, University of Torino, Torino, Italy; 5 Obstetrics, Department of Surgery, Città della Salute e della Scienza, University of Torino, Torino, Italy; 6 SSD Clinical Nutrition, Department of Clinical and Biological Sciences, ASOU San Luigi, University of Torino, Torino, Italy; 7 SS Epidemiology, Department of Clinical and Biological Sciences, ASOU San Luigi, University of Torino, Torino, Italy

**Keywords:** CKD, Low-protein Diet, Dialysis, Mortality

## Abstract

**Background:**

There is no single, gold-standard, low-protein diet (LPD) for CKD patients; the best compliance is probably obtained by personalization. This study tests the hypothesis that a multiple choice diet network allows patients to attain a good compliance level, and that, in an open-choice system, overall results are not dependent upon the specific diet, but upon the clinical characteristics of the patients.

**Methods:**

Observational study: Three LPD options were offered to all patients with severe or rapidly progressive CKD: vegan diets supplemented with alpha-ketoacids and essential aminoacids; protein-free food in substitution of normal bread and pasta; other (traditional, vegan non supplemented and tailored). Dialysis-free follow-up and survival were analyzed by Kaplan Meier curves according to diet, comorbidity and age.

Compliance and metabolic control were estimated in 147 subjects on diet at March 2015, with recent complete data, prescribed protein intake 0.6 g/Kg/day. Protein intake was assessed by Maroni Mitch formula.

**Results:**

Four hundreds and forty nine patients followed a LPD in December, 2007- March, 2015 (90% moderately restricted LPDs, 0.6 g/Kg/day of protein, 10% at lower targets); age (median 70 (19–97)) and comorbidity (Charlson index: 7) characterized our population as being in line with the usual CKD European population. Median e-GFR at start of the diet was 20 mL/min, 33.2% of the patients were diabetics. Baseline data differ significantly across diets: protein-free schemas are preferred by older, high-comorbidity patients (median age 76 years, Charlson index 8, GFR 20.5 mL/min, Proteinuria: 0.3 g/day), supplemented vegan diets by younger patients with lower GFR and higher proteinuria (median age 65 years, Charlson index 6, GFR 18.9 mL/min; Proteinuria: 1.2 g/day); other diets are chosen by an intermediate population (median age 71 years, Charlson index 6; GFR 22.5 mL/min; Proteinuria: 0.9 g/day); (*p* <0.001 for age, Charlson index, proteinuria, GFR). Adherence was good, only 1.1% of the patients were lost to follow-up and protein intake was at target in most of the cases with no differences among LPDs (protein intake: 0.47 (0.26–0.86) g/Kg/day). After adjustment for confounders, and/or selection of similar populations, no difference in mortality or dialysis start was observed on the different LPDs. Below the threshold of e-GFR 15 mL/min, 50% of the patients remain dialysis free for at least two years.

**Conclusion:**

A multiple choice LPD system may allow reaching good adherence, without competition among diets, and with promising results in terms of dialysis-free follow-up. The advantages with respect to a non-customized approach deserve confirmation in further comparative studies or RCTs.

## Background

To date, two main reasons for reconsidering on a global scale the advantages of low protein diets, as a tool to retard dialysis in younger patients and to avoid it in elderly patients, are the limited availability of dialysis in many developing countries, and the grim prognosis of elderly patients who increasingly start dialysis in developed countries [1–3].

Low protein diets had encountered mixed fortunes in the past and the term diet, often interpreted in a reductive way, has been recently challenged in favor of “nutritional therapy” in chronic kidney disease (CKD), underlining that the approach to CKD patients should be more comprehensive, beyond the restriction of one or more aliments [4, 5]. Indeed, nutritional therapies in CKD may be addressed to different goals: removing the cause or concause of CKD (such as obesity), reducing the workload on the remnant nephrons to slow progression, or stabilizing renal function so as to allow longer dialysis-free survival [3–6]. Furthermore, the term “nutritional therapies” switches the attention to therapeutic adherence or, using a more modern term, to concordance, a word that may substitute the older term compliance [7–10]. The concepts are similar but non identical: compliance implies the fact that the patient, “nudged or shoved”, has to follow the medical advice (in this case, the “best possible diet”), while a concordant attitude is centered upon patient’s preferences, and requires a flexible application, in this case, of the diet best fitting into the patient’s life [11–13].

Concordance is an appealing idea, and probably the best way to manage chronic diseases; changing towards a patient-centered attitude is however time consuming, may require a dynamic approach based upon shared choices and decisions [14–16]. As stated in a recent report of the World health Organization: “patients need to be supported, not blamed (…); adherence is simultaneously influenced by several factors (…); patient-tailored interventions are required (…); systems and providers need to develop means of accurately assessing not only adherence, but also those factors that influence it.” [17].

In the MDRD study, the largest, most controversial and fundamental RCT regarding diets in CKD, results are quite contradictory when analyzed according to “intention to treat” (demonstrating no advantage of being on a low protein diet) and “per protocol”, stratified upon urea excretion (suggesting a significant reduction of progression in patients with lower protein intake). Such a discrepancy suggests that prescribing a diet is not enough, since patients tend to follow the diet they prefer [18, 19].

Many different low-protein diet (LPD) schemes have been proposed in CKD: vegan-vegetarian or omnivorous, supplemented or non-supplemented, with protein-free commercial food or without it [20]. Schematically, most authors distinguish between “moderate” protein restriction (0.6 g of protein/Kg/day) and “very low” protein diets (0.3 g/Kg/day); each of them may be proposed by different schemas, with or without the allowance of unrestricted meals [21–27].

Flexible, personalized, holistic approaches are not easily analyzed, since by their own nature they tend to escape from the rigidity of RCTs [28–32]. Randomization to different approaches (tailored versus standardized) could highlight differences, but may be hardly feasible in single centers, in which the option of personalization is often a part of the approach to the continuum of CKD care. In such a context, observational studies are more likely to provide information on feasibility and side effects, and may overcome some of the limits of RCTs, mainly the inclusion of populations that differ from the standard ones encountered in daily practice [32, 33].

Consequently, adherence to therapy and incidence of drop-out may represent important, albeit indirect, measures of success, since we may expect that multiple-choice systems may allow easier identification of a concordant strategy.

Hence, the present study was undertaken to test the hypothesis that a multiple choice diet system in which patients are free to choose (and change) the most suitable LPD from among several options, could allow an overall good therapeutic adherence to LPDs. We analyzed a large single-center cohort of on-diet patients (449 patients, 847 patient-years of observation, in which diets, mainly with a moderate protein restriction, and controls follow a personalized schedule of prescriptions and check-ups, adapted to the various clinical situations [20, 33–36].

## Methods

### Study setting; patient selection and inclusion criteria

The study was carried out at the out-patient Nephrology Unit of the San Luigi Hospital, University of Torino, Italy. The Unit started its activity on 1^st^ December, 2007, and by 31^st^ March, 2015, over 4000 patients had been evaluated.

CKD was defined according to the usual definitions of the K/DOQI clinical practice guidelines; e-GFR was assessed by the CKD-EPI equation, and compliance to the protein intake by the Maroni-Mitch formula [37–39].

In the study setting, an LPD is routinely proposed by the nephrologist to all pre-dialysis patients with CKD stages 4-5, and to those with rapid progression of CKD stage 3 and/or with refractory nephrotic syndrome, in the absence of contraindications, as elsewhere described more in detail [33].

### Diets and controls

Over time, the approach to LPDs shifted from the proposal of two main LPDs (0.6 g/Kg/day: vegan-vegetarian supplemented with alpha-ketoacids and aminoacids (LPD-AK) and with protein-free commercial food (LPD-PFF)), which were eventually merged into one very-low protein diet (vLPD), to a more integrated choice including different combinations of the previous diets and vegan non-supplemented diets [20, 33–36] (Table 1).

**Table 1:** The various low-protein diets offered in our Unit and included in this study

Type of diet	Protein restriction (g/Kg of body weight per day)	Main features	Notes
“Traditional”	0.6–0.8 g/Kg/day; mixed protein (animal and plant derived)	Modulated upon quantity of usual food; mainly based on the traditional Italian regional cuisines.	Often corresponds to what elderly patients already follow, in particular if they cook their own food from raw ingredients.
Vegan	0.6–0.8 g/Kg/day; vegetable protein	Protein intake in unrestricted vegan diets is on the average 0.7–0.9 g/Kg/day; due to the different bioavailability, a 0.7 diet roughly corresponds to a 0.6 mixed protein diet	This diet is based upon the integration of cereals and legumes at each meal, thus ensuring complementarity in amino acids. The 1–3 unrestricted meals per week are also crucial for this goal.
Vegan supplemented (moderate restriction)	0.6 g/Kg/day; vegetable protein, supplemented with amino- and keto-acids (Alfa-kappa or Ketosteril)	Based upon forbidden (animal origin) and allowed (all other) food. Animal-derived food is allowed in unrestricted meals. Supplementation with Alfa-kappa or Ketosteril pills is tailored upon nutritional status and clinical situation (1:8–1:10 Kg BW)	The supplements of amino- and ketoacids ensure protein complementarity without need to choose among plant derived food.
Protein-free food	0.6 g/Kg/day; mixed protein	Protein-free pasta, bread and other carbohydrates substituting for the usual bread, pasta or rice.	Since carbohydrates are the basis of the Mediterranean cuisine, their substitution allows subjects to easily reach a 0.6 g/Kg/BW diet
Very low-protein supplemented vegan diet	0.3 g/Kg/day; vegetable protein only, supplemented with Alfa-kappa or Ketosteril, with protein-free food	This diet is also based upon forbidden (animal origin) and allowed (all other) food. Animal-derived food is allowed only in “free meals” (usually no more than 1 per week) Supplementation with Alfa-kappa or Ketosteril pills is higher (1:5 Kg BW). Carbohydrates are mainly protein-free.	This diet merges the concepts of vegan supplemented and protein-free food. It is demanding and requires compliance to the high number of pills employed in supplementation. It is not prescribed as a “first line” diet.
Tailored solutions	Usually 0.6 g/Kg/day, vegetable or mixed	These solutions employ different combinations of protein-free food, vegan diets and supplementation.	The main reason for prescribing these diets is to take into account patients’ needs or preferences: an example: alternating vegan-supplemented meal and protein-free food.

*BW* body weight

All diets share a simplified and flexible approach aimed at facilitating integration into daily life. The LPD prescription is qualitative: it is based on allowed and forbidden foods, with 1-3 unrestricted meals per week (depending on the patients’ preferences and kidney function) [33]. The first diet prescribed in our Unit is a moderately restricted diet (on average 0.6 g/Kg/day of protein) and only after a successful trial, in selected cases a 0.3 diet is prescribed.

In our region, both protein-free food and Ketosteril or Alfa-kappa are provided free of charge (maximum expenditure for protein-free food: 120 euro per month).

Daily energy intake is aimed at 30–35 kcal/Kg/day; caloric intake is calculated on the basis of a diet journal that is kept for 1–3 weeks. In elderly patients a caloric intake of 25–30 kcal/Kg/day was considered acceptable, provided that weight, albumin and bioimpedence measures remained stable.

Supplementation with calcium, vitamin D, folic acid, vitamin B12, iron and erythropoietin is tailored according to blood levels on the basis of the usual indications. Biochemical exams and routine visits are scheduled every 1–2 months in stable subjects and up to once a week for patients with severe metabolic derangements or when GFR drops to below 7–10 mL/min. Dialysis start is decided on the basis of the clinical picture (including symptoms such as weight loss, nausea, malnutrition, restless leg syndrome, poorly controlled hypertension); GFR, urea levels, water and acid-base balance, calcium-phosphate-PTH balance, anemia and albuminemia are taken into account in the overall evaluation of the patient.

The simplified diets were originally designed and prescribed by the nephrologist; between 2007 and 2012, consultation with a dietitian was available only for complex cases (overweight, underweight; specific situations such as celiac disease or food allergy). Since 2013, a part-time dietitian was enrolled in the team, thus ensuring systematic counseling for all on-diet patients; her support leaded also to an increase in personalized dialysis schedules (LPD-Other).

### Biochemical data

Biochemical parameters are always assessed in the patient’s laboratory of choice, and for about 70% of subjects this means the General Laboratory of our hospital. Regularly controlled biochemical data included serum creatinine (mg/dL), BUN (mg/dL), GFR (CKD-EPI), proteinuria (g/day); HCO3 (mmol/L), albumin (g/dL), parathyroid hormone (PTH, pg/mL), protein intake (Maroni Mitch formula [39]).

For the sake of uniformity in the dosage methods, only the results of tests performed in the General Laboratory of the hospital in the last period of study were included in the analysis of compliance. Furthermore, we standardized our 24 h urine collection (at least 48 h from an unrestricted meal) only in 2014–2015; therefore, this analysis was carried out in prevalent patients at March 31^st^, 2015, considering the data available in our laboratory, updated within 3 months (147 cases).

### Statistical analysis

A descriptive analysis was performed as appropriate (median and range for non-parametric data, mean and standard deviation for parametric distribution), ANOVA, Kruskal-Wallis, Mann-Whitney, and *t*-test were performed according to standard indications for continuous variables, while risks, rates and proportions were compared using Chi-square and Fisher's exact test.

Survival analysis was performed according to Kaplan Meier also in order to identify the relevant covariates to be entered into a multivariate analysis. The following covariates were analyzed: type of diet (intention to treat); moderate protein restriction; vegan supplemented; with protein-free food; other diets.

Age was dichotomized at 65 years, or analyzed with the following strata (<64; 65–75; > = 75); comorbidity was weighted by the Charlson index (dichotomized at 7) [40]. Significance of the differences was tested by the log-rank test and Wilcoxon test.

Three outcomes were considered: dialysis (renal death), mortality, combined outcome (death or dialysis). The beginning of the observation period was either the start of the diet or referral to the Unit for patients who were already on LPDs. The end of observation was the end of the diet. A separate analysis was performed starting with the observation at the first recording of GFR < 10 or <15 mL/min (considered “late-conventional” and “early” start of dialysis, respectively).

### Multivariate and stratified analysis

Survival analysis: Cox analysis was performed considering the following covariates: Charlson index (dichotomized at 7); type of diet (first diet, following an intention to treat analysis; due to the heterogeneity of the “other” diets, we included only the two main ones: LPD-AK and LPD-PFF in the analysis); GFR (dichotomized at 30 mL/min) and proteinuria (dichotomized at 1 g/day) at the start of the diet. Age, which is included in the Charlson index, was not entered in the model due to co-linearity.

For the analysis of dialysis start, to account for the attrition bias of death versus the risk of starting dialysis (competitive mortality), we selected a homogeneous group of subjects with regard to age, comorbidity, and kidney function (age > =65–<80 years; Charlson index > =5–<12, baseline proteinuria <3 g/day, initial GFR > =15–<30 mL/min). Diet, Charlson index and GFR were entered into the Cox analysis.

### Ethical issues

Informed consent was obtained for anonymous management of the clinical data. Individual consent for publication was not needed for this study, dealing with overall data and not with single cases.

The observational study design (PROTEREne: PROTEin REductio to protect the REins) based on standard clinical practice, that includes patients with severe CKD, was approved by the Ethics Committee of the San Luigi Hospital, University of Torino (Delibera 22, 18 January 2013, protocol 000037).

## Results

### Baseline data

Patients who opted for different diets have different baseline characteristics (Table 2). Patients who choose a low protein vegan supplemented diet (LPD-KA) are significantly younger, with lower comorbidity and higher prevalence of glomerulonephritis, nephrotic proteinuria and severely impaired GFR, as compared to patients who select an LPD based upon the substitution of the usual carbohydrates with protein-free food (LPD-PFF). The latter are significantly older, their kidney diseases are more often linked to cardiovascular impairment, and glomerulonephritides are seldom encountered. Followers of the “other” options, more recently implemented in the system, including vegan or Mediterranean diets, are in the intermediate age and comorbidity group (Table 2).

**Table 2:** Baseline characteristics of the population, on the basis of the first diet

First diet	Vegan supplemented	With protein- free food	Other	All cases	*p* Among groups
n	215	159	75	449	
Males (%)	144 (67.0%)	98 (61.6%)	34 (45.3%)	276 (61.5%)	0.004
Females (%)	71 (33.0%)	61 (38.4%)	41 (54.7%)	173 (38.5%)	
Age: median (min-max)	65 (19–86)	76 (26–97)	71 (23–88)	70 (19–97)	<0.001
Age over 65 (%)	100 (46.5%)	132 (83%)	42 (56%)	274 (61%)	<0.001
Age over 80 (%)	15 (7.0%)	44 (27.7%)	14 (18.7%)	73 (16.3%)	<0.001
Charlson: median (min-max)	6 (2–12)	8 (2–13)	6 (2–12)	7 (2–13)	<0.001
Charlson > =7 (%)	91 (42.3%)	118 (74.2%)	37 (49.3%)	246 (54.8%)	<0.001
Charlson > =10 (%)	17 (7.9%)	42 (26.4%)	12 (16.0%)	71 (15.8%)	<0.001
Diabetes (%)	59 (27.4%)	70 (44%)	20 (26.7%)	149 (33.2%)	0.001
Cardiopathy (%)	79 (36.7%)	100 (62.9%)	29 (38.7%)	208 (46.3%)	<0.001
Neoplasia (%)	41 (19.1%)	33 (20.8%)	23 (30.7%)	97 (21.6%)	0.104
sCreatinine (mg/dL) median (min-max)	3.05 (0.6–16)	2.65 (1.0–7)	2.49 (0.6–6.7)	2.8 (0.6–16)	<0.001
eGFR-EPI (mL/min) median (min-max)	18.9 (3–126)	20.5 (6.6–73)	22.5 (5.7–127)	20.0 (3–127)	0.014
GFR <15 mL/min at enrolment n (%)	80 (37.6%)	32 (20.1%)	13 (17.3%)	125 (28%)	<0.001
GFR <10 mL/min at enrolment n (%)	29 (13.6%)	10 (6.3%)	5 (6.7%)	44 (9.8%)	0.038
Proteinuria (g/day) median (min-max)	1.2 (0.1–10)	0.3 (0.1–7)	0.9 (0.1–11)	0.8 (0.1–11)	<0.001
Proteinuria > = 1 g/day (%)	125 (58.7%)	41 (26.1%)	36 (48.0%)	202 (45.4%)	<0.001
Proteinuria > = 3 g/day (%)	50 (23.5%)	18 (11.5%)	14 (18.7%)	82 (18.4%)	0.013
Glomerulonephritis-systemic disease (%)	67 (31.2%)	12 (7.5%)	16 (21.3%)	95 (21.2%)	<0.001
Nephroangiosclerosis and/or diabetes (%)	100 (46.5%)	128 (80.5%)	37 (49.3%)	265 (59.0%)	<0.001
ADPKD (%)	13 (6.0%)	4 (2.5%)	7 (9.3%)	24 (5.3%)	0.079

*Charlson* Charlson’s comorbidity index, *E-GFR EPI* GFR according to the CKD-EPI equation, *ADPKD* autosomal dominant polycystic kidney disease

### Main outcomes

The main outcomes are unevenly distributed, related to the first diet that was chosen (Table 3). Half of the patients are still on an LPD at the time of this study (225 patients); thanks to-choose the flexible approach only few patients discontinued the diet or were lost to follow-up (5 cases, 1.1%).

**Table 3:** Main outcomes: distribution based on the first diet

First diet	Vegan supplemented	With protein-free food	Other	All cases	*p* Among groups
Continues n (%)	86 (40.0%)	81 (50.9%)	58 (77.3%)	225 (50.1%)	<0.001
Discontinued n(%)	6 (2.8%)	2 (1.3%)	0	8 (1.8%)	
Transferred n (%)	4 (1.9%)	1 (0.6%)	1 (1.3%)	6 (1.3%)	
Lost to follow-up n (%)	1 (0.5%)	4 (2.5%)	0	5 (1.1%)	
On dialysis n (%)	83 (38.6%)	12 (7.5%)	10 (13.3%)	105 (23.4%)	
Dead n (%)	35 (16.3%)	59 (37.1%)	6 (8.0%)	100 (22.3%)	
Overall follow-up (years)	375.67	335	136.5	847.17	0.124

9 further deaths were recorded in the first year after discontinuation of the diet; all occurred on dialysis (on further 94.4 years of observation)

As expected by the baseline distribution, death is the most common cause of discontinuation on LPD-PFF (patients are about 10 years older and with higher comorbidity); dialysis start is the most common cause of discontinuation in patients who start on an LPD-KA, in keeping with a longer life-expectancy of these younger patients, one third of whom are affected by glomerular or systemic diseases. The heterogeneous group of patients who chose other schedules lies between these first two as for age and comorbidity; however, serum creatinine is lower and e-GFR higher in this subset of cases, reflecting a slow and cautious integration of the new LPD approaches in the context of a well established use of the previous two schemes (LPD-KA and LPD-PFF).

**Fig. 1:**
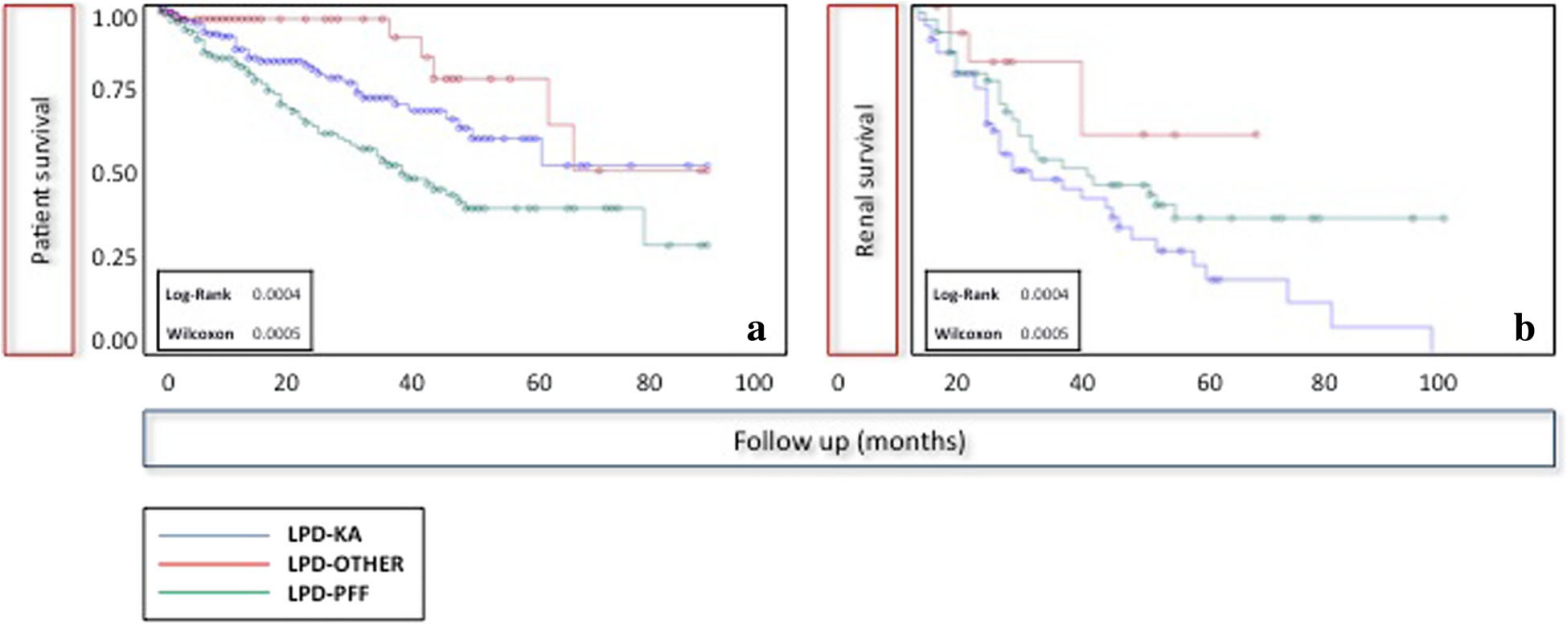
Patient survival and “renal survival” according to the diet chosen

The attrition bias due to the competition between mortality and dialysis start is highlighted in Fig. 1: the patients with higher mortality (higher Charlson index, older age) have a lower risk of dialysis start. Accordingly, the two main diets, LPD-PFF and LPD-KA, have opposite patterns with respect to mortality (higher in the former) and dialysis (higher in the latter).

Cardiovascular diseases are the most common cause of death, as expected by the comorbidity burden (patients who died: median Charlson index at start of the diet: 9) (Table 4).

**Table 4:** Characteristics of the patients who died, stratified on the basis of the first diet

First diet	Vegan supplemented	With protein- free food	Other	All cases	*p* Among groups
n	35	59	6	100	
Males/Females (%)	29 (82.9%) 6 (17.1%)	38 (64.4%) 21 (35.6%)	5 (83.3%) 1 (16.7%)	72 (72.0%) 28 (28.0%)	0.128
Age at start of the diet median (min-max)	71	78	70	74	0.003
Charlson index median (min max)	9 (6–12)	9 (5–12)	8 (2–11)	9 (2–12)	0.794
Charlson > =7 (%)	30 (85.7%)	53 (89.8%)	4 (66.7%)	87 (87.0%)	0.264
Charlson > =10 (%)	9 (25.7%)	19 (32.2%)	2 (33.3%)	30 (30.0%)	0.789
Diabetes (%)	17 (48.6%)	30 (50.8%)	3 (50.0%)	50 (50.0%)	0.977
Cardiopathy (%)	27 (77.1%)	49 (83.1%)	4 (66.7%)	80 (80.0%)	0.552
Neoplasia (%)	9 (25.7%)	12 (20.3%)	1 (16.7%)	22 (22.0%)	0.788
s Creatinine (mg/dL) median, (min-max)	3.0 1.6–5	2.5 1.1–5.9	3.65 2.6–4.3	2.90 1.1–5.9	0.002
eGFR-EPI (mL/min) median, (min-max)	18.9 10.9–44.5	20.8 7.7–73.1	16.0 10.3–17.7	20.0 7.7–73.1	0.022
Proteinuria (g/day) median, (min-max)	0.5 0.1–4.6	0.2 0.1–6.3	0.2 0.1–1.0	0.35 0.1–6.3	0.111
Proteinuria > = 1 g/day (%)	12 (34.3%)	13 (22.8%)	1 (16.7%)	26 (26.0%)	0.410
Glomerulonephritis-systemic disease (%)	3 (8.6%)	1 (1.7%)	0	4 (4%)	0.226
Nephroangiosclerosis and/or diabetes (%)	30 (85.7%)	52 (88.1%)	4 (66.7%)	86 (86%)	0.352
Main causes of death					
Cardiovascular (%)	16 (45.7%)	29 (49.1%)	2 (33.3%)	47 (47%)	0.590
Neoplasia (%)	4 (11.4%)	3 (5.1%)	1 (16.7%)	8 (8%)	
Lung disease (%)	1 (2.8%)	4 (6.7%)	0	5 (5%)	
Infection (%)	4 (11.4%)	5 (8.5%)	0	9 (9%)	
Other (%)	6 (17.1%)	5 (8.5%)	2 (33.3%)	13 (13%)	
Unknown (%)	4 (11.4%)	13 (22.1%)	1 (16.7%)	18 (18%)	

Patients who start dialysis are significantly younger and with less comorbidity than those who die (Tables 4 and 5). Of note, only 3 patients started unplanned dialysis (in 1 case because of the rupture of a polycystic kidney and in 2 because of fluid overload; the two latter subjects already had an AV fistula and dialysis was started on the day of a planned clinical visit) (Table 5).

**Table 5:** Characteristics of the patients who started dialysis (listed on the basis of the first diet)

First diet	Vegan supplemented	With protein- free food	Other	All cases	*p* Among groups
n	83	12	10	105	
Males/Females (%)	52 (62.7%) 31 (37.3%)	8 (66.7%) 4 (33.3%)	4 (40.0%) 6 (60.0%)	64 (61.0%) 41 (39.0%)	0.348
Age at start of diet: median (min max)	63 (23–86)	72 (59–86)	69.5 (29–83)	64 (23–86)	0.009
Age over 65 (%)	32 (38.6%)	9 (75.0%)	6 (60.0%)	47 (44.8%)	0.036
Age over 80 (%)	4 (4.8%)	2 (16.7%)	1 (10.0%)	7 (6.7%)	0.278
Charlson index median (min max)	6 (2–11)	7.5 (5–12)	8 (4–10)	6 (2–12)	0.001
Charlson > =7 (%)	30 (36.1%)	9 (75.0%)	8 (80.0%)	47 (44.8%)	0.003
Charlson > =10 (%)	5 (6.0%)	3 (25.0%)	1 (10.0%)	9 (8.6%)	0.089
Diabetes (%)	23 (27.7%)	7 (58.3%)	4 (40.0%)	34 (32.4%)	0.091
Cardiopathy (%)	26 (31.3%)	6 (50.0%)	6 (60.0%)	38 (36.2%)	0.117
Neoplasia (%)	16 (19.3%)	2 (16.7%)	6 (60.0%)	24 (22.9%)	0.013
s Creatinine (mg/dL) median, (min-max)	4.5 (1.5–16.0)	3.3 (2.3–6.4)	3.6 (2.5–6.7)	4.2 (1.5–16.0)	0.492
eGFR-EPI (mL/min) median, (min-max)	12.8 (3.0–44.6)	16.2 (7.7–25.5)	15.1 (5.7–19.7)	13.2 (3.0–44.6)	0.885
Proteinuria (g/day) median, (min-max)	1.5 (0.1–9)	1.4 (0.7–5.7)	3.3 (0.1–11)	1.5 (0.1–11)	0.407
Proteinuria > =1 g/day (%)	55 (67.9%)	8 (66.7%)	7 (70.0%)	70 (68.0%)	0.986
Proteinuria > =3 g/day (%)	21 (25.9%)	4 (33.3%)	5 (50.0%)	30 (29.1%)	0.270
Glomerulonephritis-systemic disease (%)	26 (31.3%)	2 (16.7%)	2 (20.0%)	30 (28.6%)	0.472
Nephroangiosclerosis and/or diabetes (%)	37 (44.6%)	10 (83.3%)	5 (50.0%)	52 (49.5%)	0.043
ADPKD (%)	7 (8.4%)	0 (0.0%)	2 (20.0%)	9 (8.6%)	0.247
Type of dialysis (% HD)	71 (85.5%)	10 (83.3%)	10 (100%)	91 (86.6%)	0.457
dialysis in emergency n (%)	3 (3.6%)	0	0	3 (2.8%)	0.630

### Patient survival: Cox analysis

According to Cox analysis, which was performed considering only the two most frequent diets with comparable follow-up (LPD-KA and LPD-PFF, both with over 300 patient-years of observation), survival is significantly affected by Charlson index, but neither the diet nor kidney function or proteinuria at baseline retained a significant effect (Table 6).

**Table 6:** Crude and adjusted HRs of mortality, by diet, Charlson index, proteinuria, and GFR (Cox analysis) (LPD-KA and LPD-PFF)

	n/N	Crude RR (95% CIs)	*p* value	Adjusted HR (95% CIs)	*p* value
Diet					
LPD-KA	37/215	1 (−)	<0.0001	**1 (−)**	0.5264
LPD-PFF	59/159	2.16 (1.51–3.08)		**1.15 (0.75–1.75)**	
Charlson index					<0.0001
<7	12/165	1 (−)	<0.0001	**1 (−)**	
≥7	84/209	5.53 (3.13–9.77)		**6.11 (3.28–11.36)**	
Proteinuria					
*(g/day)*			<0.0001		0.2107
< 1	69/207	1 (−)		**1 (−)**	
≥ 1	27/167	0.49 (0.33–0.72)		**0.75 (0.48–1.18)**	
GFR (ml/m)					0.2425
< 30	78/291	1 (−)	0.3436	**1 (−)**	
≥ 30	18/83	0.81 (0.72–1.27)		**0.74 (0.44–1.23)**	

*GFR-EPI* Glomerular filtration rate (EPI formula), *RR* Relative risk, *HR* Hazard ratio

statistically significant OR in bold

### Dialysis start: the effect of the diet in selected subsets of homogeneous cases

**Fig. 2:**
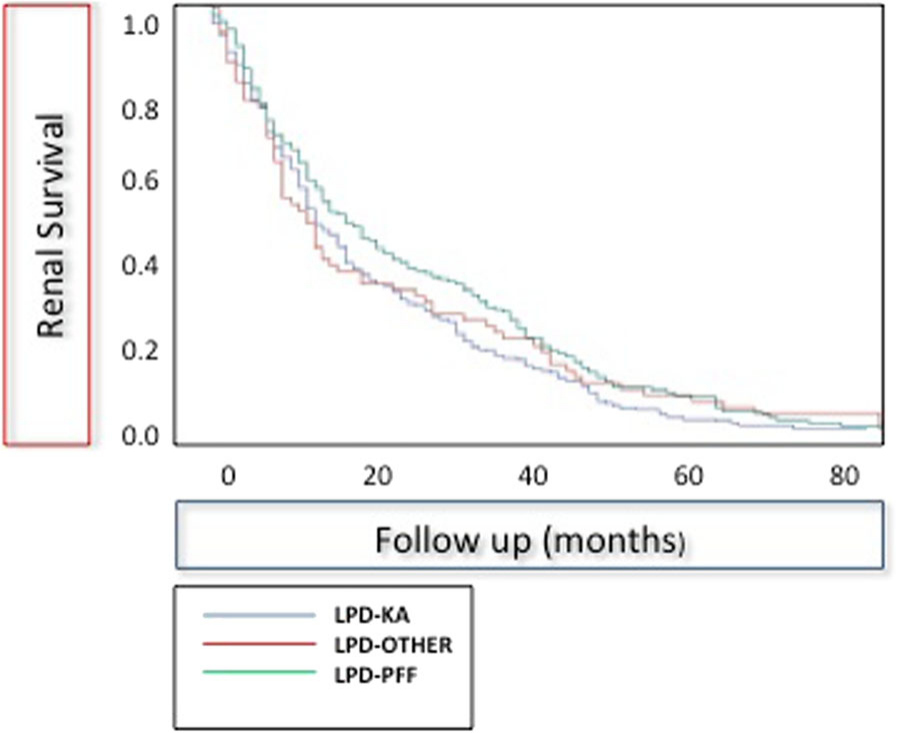
Combined outcome of patient and renal survival in a selected population: age > =65 and <80 years; Charlson index >4 and <12, baseline proteinuria < 3 g/day and GFR at start between 15 and 30 mL/min

Table 7 summarizes the analysis performed on a selected population (age > =65 and <80 years; Charlson index >4 and <12, baseline proteinuria < 3 g/day and GFR at start between 15 and 30 mL/min), chosen to try to overcome the attrition bias of mortality, given the baseline differences by self-allocation of different patients to different diets (Table 2). According to this analysis, there is no difference according to LPD, and only proteinuria retains a significant effect on renal survival (Fig. 2: outcome ESRD or death in the selected population).

**Table 7:** Crude and adjusted HRs of dialysis start by main diet (LPD-KA, LPD-PFF), Charlson index, proteinuria, and GFR (Cox analysis) in the population: age > =65–<80 years; Charlson index > =5 < 12; proteinuria <3 g/day GFR at start > =15 < 30 mL/min

	n/N	Crude RR (95% CIs)	*P* value	Adjusted HR (95% CIs)	*P* value
Diet			0.19		0.2326
LPD-KA	6/32	(1−)		(1−)	
LPD-PFF	4/46	0.46 (0.14–1.51)		0.43 (0.11–1.72)	
Charlson index			0.38		0.7511
<7	2/25	(1−)		(1−)	
≥7	8/53	1.89 (0.43–8.25)		1.31 (0.25–6.93)	
Proteinuria (g/day)			0.002		0.0303
<1	3/55	(1−)		(1−)	
≥1	7/23	5.58 (1.58–19.71)		5.13 (1.17–22.55)	

*RR* Relative risk, *HR* Hazard ratio

### Compliance and metabolic control

Table 8 reports the main biochemical data in a prevalent subset of the on-diet population (on a 0.6 g/Kg/day diet) as of March 31^st^, 2015, who underwent metabolic and renal functional assessment within three months in the general laboratory of our hospital (147 patients, out of 209 prevalent patients on a 0.6 g/Kg/day of protein diet on follow-up at the same date). The general characteristics of this subset are as expected from our enrollment criteria: median e-GFR below 30 mL/min, about one fourth of the patients with e-GFR <15 mL/min. Compliance to the diet (0.6 g/Kg/day) assessed on a 24-h urine collection was very good (median 0.47 g/Kg/day, performed at least 48 h after the last unrestricted meal) and more than half of the patients were slightly below the prescribed intake. Moreover, serum albumin was within the normal range in most cases, and the 11 patients with serum albumin <3 g/dL were characterized by low Charlson index (median 4, range 2–9) and high proteinuria (median 5.6, min-max: 1.1–10.9 g/day), suggesting that this was most likely an effect of the kidney disease and not of malnutrition. Furthermore, parathyroid hormone and bicarbonate levels were on target in most cases, and only 5 patients (3 of whom had severe cardiac disease and high need for diuretics) had a BUN above 100 mg/dL.

**Table 8:** Compliance and main biochemical data at the (last updating): 147 on-diet patients who underwent complete biochemical profiling by March 31^st^, 2015 (with a complete biochemical profile performed at the san Luigi General Laboratory)

Diet history	LPD-KA only	LPD-PFF only	Other only	More than one diet	All patients	*p* Among groups
n	54	46	32	15	147	
Males/Females	39 (72.2%) 15 (27.8%)	25 (54.3%) 21 (45.7%)	13 (40.6%) 19 (59.4%)	8 (53.3%) 7 (46.7%)	85 (57.8%) 62 (42.2%)	0.032
sCreatinine mg/dL: median (min-max)	2.7 1.2–8.1	3.1 1.3–11.8	2.4 1.3–11.5	2.9 1.4–8.0	2.8 1.2–11.8	0.106
Creatinine Cl. (mL/min) median (min-max)	27 6–103	18 6–59	31 10–66	13 5–57	22 5–103	0.024
Proteinuria g/day: median, (min-max)	1.25 0.1–10.9	0.33 0.1–3.4	0.86 0.1–6.4	1.1 0.1–9.8	0.79 0.1–10.9	0.004
Proteinuria ≥ 1 g/day(%)	29 (53.7%)	15 (32.6%)	16 (50.0%)	8 (53.3%)	68 (46.3%)	0.162
s-albumin g/dL: median (min-max)	3.71 1.80–4.70	3.79 3.10–5.00	3.87 2.50–4.60	3.50 2.42–4.10	3.76 1.80–5.00	0.165
Albumin <3 g/dL (%)	7 (13.0%)	0	1 (3.1%)	3 (20.0%)	11 (7.5%)	0.016
PTH pg/mL: median (min-max)	77 20–333	112 34–848	104 36–705	25 21–280	99.7 20.0–848.0	0.027
PTH >300 pg/mL(%)	2 (3.8%)	5 (10.9%)	2 (6.3%)	2 (14.3%)	11 (7.6%)	0.439
BUN mg/dL, median (min-max)	44.5 11–98.5	53 9.5–111.5	38.5 17–93.5	46.5 23–100	46.5 9.5–111.5	0.076
BUN > =100 mg/dL	0	3 (6.5%)	0	2 (13.3%)	5 (3.4%)	0.031
HCO3 median, mg/dL)	25.1 14.3–31.0	26.2 17.7–39.0	25.6 17.0–34.0	24.6 20.8–28.0	25.6 14.3–39.0	0.244
HCO3 < 20 mg/dL	5 (9.8%)	3 (7.0%)	3 (10.0%)	0	11 (8.1%)	0.710
Protein intake (Mitch formula) g/Kg/day: median (min-max)	0.48 0.36–0.76	0.44 0.29–0.86	0.52 0.32–0.83	0.46 0.26–0.67	0.47 0.26–0.86	0.002
Protein <0.6 g/Kg/day	29 (54.3%)	35 (76.1%)	13 (40.6%)	10 (66.7%)	87 (59.6%)	0.013
Protein ≥0.8 g/Kg/day	0	1 (2.2%)	2 (6.3%)	0	3 (2.0%)	0.233

The 48 patients who did not complete biochemical profiling in our laboratory in the last 3 months were older and had a higher Charlson index, but with similar creatinine, e-GFR, and diet history as compared to the whole on-diet population, thus confirming the advantage of tailoring the check-ups also considering the patient’s needs

## Discussion

The main point of interest in our study regards the multiple-choice approach developed, which indirectly demonstrated the relevance of patient’s preferences: when patients are given various options, different categories of individuals choose different diets (Tables 1 and 2). These data are in keeping with previous studies by our group that suggested, on a smaller scale, that LPD choice reflected the patients’ characteristics: elderly patients, less willing to change their dietary habits, prefer diets based upon the substitution of regular carbohydrates (mainly bread and pasta in Italy, which contain 11–12% of protein) with protein-free food, while younger patients prefer a simplified scheme of supplemented vegan/vegetarian diets, which are more compatible with their “social life” [20, 25, 33]. Other experts report similar data on diets based upon protein-free food, suggesting that such an option, which is free of charge in our country, should also be considered in other settings [14–16, 23, 26, 41, 42].

While only larger multicenter studies, comparing (and eventually randomizing) different approaches to protein restriction in CKD may definitely highlight the specific features, advantages and draw-backs of a multiple-choice dietary approach, flexibility may be the reason why only a minority of the patients discontinued LPDs (1.8%), or was lost to follow-up (1.1%), while about 10% of the subjects changed at least one diet over time (Table 3). Likewise, it may offer an indirect explanation to the good compliance and metabolic control observed: an up-date involving a sample of 147 on-diet prevalent stable subjects with complete data showed that protein intake was even lower than recommended i.e., 0.47 versus 0.6 g/Kg/day, with satisfactory nutritional data and metabolic data (Table 8).

Taking into account the baseline differences, it is not surprising that both renal and patient survival differ according to the diet chosen, here analyzed as “intention to treat”, sorting patients according to the first diet (Fig. 1). Patient survival was the lowest on the diet based upon protein-free food, as expected in a significantly older with a higher Charlson index (8, meaning a survival probability of 35% at two years) [40]. The independence of survival form the diet choice is confirmed by Cox analysis, that confirms the importance of Charlson index (which includes the effect of age) and fails to identify an effect of diet or of severity of the kidney disease (Tables 4, 6).

Mortality represents a considerable attrition bias for dialysis start, and “renal survival” was the lowest in the younger population (Fig. 1); however, the differences disappear in survival and multivariate analysis, when a population at comparable risk of death is selected (Table 7, Fig. 2).

Like all clinical studies, ours has merits and limits. The merit is the novelty and the fact that it includes one of the largest, currently available survival analyses regarding a cohort of patients treated with different LPDs, mainly moderately restricted. It proposes to analyze LPDs as the different dialysis choices i.e., a system and not the sum of single treatments, in which patients are left free to self-allocate to the treatment(s) that best fit into their daily life, suggesting that in such a context differences among results reflect the patients’ characteristics and not the treatment options [43].

Our study is not a randomized trial; while this choice may better reflect the clinical reality, it lacks the strength of RCTs, and does not allow comparing with other models of care (not flexible, or including one “best” diet only) [28–31].

Besides the limitations shared by all non randomized approaches, the progressive development of our system doesn’t allow sound comparison with the “tailored” diet options that were more recently developed in our Unit, whose promising results need to be validated over al longer follow-up.

The promising results, together with the limits of this first experience highlight the need of a prospective multicentric analysis including various LPDs and different diet-systems, that could be compared or to identify the best strategies of attaining compliance.

## Conclusions

Different CKD patients choose different diets; when several options are present, a relevant percentage of patients experience more than one option; in such a system, drop-out and loss to follow-up are exceedingly rare, and compliance is remarkably good. While only comparing different approaches, at best with a randomized trial, can highlight the specific merits and drawbacks of such a system, the results obtained suggest that an option among various LPDs may be offered to CKD patients without “competition” between different diets, and with good clinical results.
